# Effect of Gender on the Outcomes of ST-Elevation Myocardial Infarction at a Tertiary Care Hospital in Riyadh, Saudi Arabia

**DOI:** 10.7759/cureus.10118

**Published:** 2020-08-29

**Authors:** Mohammed S Alharbi, Bander K Alanazi, Ibrahim A Alquhays, Nawaf A Alhamied, Abdullah Al Shimemeri

**Affiliations:** 1 Medicine, Qassim University, Buraiydah, SAU; 2 Medicine, King Faisal University, Al Ahsa, SAU; 3 Internal Medicine, King Faisal University, Al Ahsa, SAU; 4 Medicine, King Saud Bin Abdulaziz University for Health Sciences, Riyadh, SAU; 5 Internal Medicine: Critical Care, King Saud Bin Abdulaziz University for Health Sciences, Riyadh, SAU

**Keywords:** st elevation myocardial infarction, myocardial reperfusion, complications

## Abstract

Objective

This study aimed to evaluate the impact of gender on the outcomes among ST elevation myocardial infarction patients at King Abdulaziz Medical City in Riyadh, Saudi Arabia.

Methods

This retrospective study analyzed the data of 900 patients (770 males and 130 females) admitted between January 2016 and December 2018 diagnosed with ST-elevation myocardial infarction (STEMI). We recorded the baseline characteristics, comorbidities, treatment, complications, and mortality for all patients, and compared these data between female and male patients.

Results

The baseline characteristics: BMI and age were higher in females and were statistically significant (p = 0.0001). We found a higher incidence of heart failure in females than in males which was statistically significant (p = 0.0010). In addition, the mortality rate was higher in female than in male patients, although this difference was not statistically significant (p = 0.3850).

Conclusion

In conclusion, despite the advances in the technology and the use of novel reperfusion therapies females were associated with poorer outcomes after adjustment of the baseline characteristics and risk factors. In other words, heart failure, mitral regurgitation, and arrhythmias were higher in females with significant p values.

## Introduction

Globally, around 3 million people sustain ST-elevation myocardial infarction (STEMI) annually, while around 4 million people suffer from non-ST elevation myocardial infarction (NSTEMI) [1]. The rate of STEMI in men is twice as high as that in women [2]. Continuous advancements in reperfusion therapy have resulted in a significant decline in the mortality and related complications in patients with STEMI [3,4]. Multiple studies have investigated the risk factors of mortality, complications, and prognostic factors after STEMI and the results in some studies showed that female gender may adversely affect the clinical outcome in STEMI and it is associated with higher mortality and more complications [5,6]. While the others attributed the difference in the baseline characteristics to be the main reason beyond the poorer outcome rather than the gender itself [7,8].

The structure of the left ventricle differs between the genders, and females are more prone to heart failure due to concentric remodeling despite preserved LV ejection fractions [9,10]. Women show a higher incidence of mechanical failures, such as mitral regurgitation and ventricular septal rupture [11]. In most studies, female patients with STEMI also had a higher age and concurrent disease burden, such as diabetes, hypertension, etc., than males [5,6,12,13].

It is not clearly understood whether left ventricular systolic dysfunction after myocardial infarction is responsible for the poorer clinical outcomes and higher rates of complications in women [14].

However, other studies did not find a correlation between gender and clinical outcome, they attributed the higher mortality in female patients to a higher age and Killip class at the time of admission, other concurrent diseases, and confounding factors [11,13,15]. The health status of female patients at the time of myocardial infarction has also been found to affect the outcomes [16].

Few recent studies have studied gender differences that may affect the prognosis after STEMI in female patients in details. Given the ambiguity in the literature, we need to understand gender-based variations so that tailored therapeutic approaches can be designed. Further, in view of the financial and socio-economic burden of myocardial infarction and coronary disease, it is important to study the mechanisms and the underlying causes that affect clinical outcomes in women. This study aimed to evaluate the impact of gender on the outcomes among ST elevation myocardial infarction patients at King Abdulaziz Medical City in Riyadh, Saudi Arabia.

## Materials and methods

This study was approved by the ethics committee of King Abdullah International Medical Research Center (No. RSS19/038/R) and conformed to the ethical principles outlined in the Declaration of Helsinki. We performed a retrospective chart review of all adult patients (n = 900) with a final diagnosis of STEMI admitted to King Abdulaziz Medical City, Riyadh, Saudi Arabia, between January 2016 and December 2018.

We extracted the following data from the hospital’s BestCare database using a customized data collection sheet: demographic data such as age, gender, nationality, height, weight, body mass index (BMI). Moreover, data regarding comorbidities such as hypertension, hyperlipidemia, and diabetes were collected as well as risk factors such as the history of smoking, renal failure, chronic obstructive pulmonary disease, and stroke. We also recorded the cardiac troponins, creatine kinase (CK), CK-MB isoenzyme, electrocardiography (ECG) findings, and echocardiographic data which included: left ventricular ejection fraction (LVEF), significant mitral regurgitation, and any other post-myocardial infarction abnormalities. We further noted the details of treatment, such as coronary artery bypass graft, percutaneous coronary intervention, intravenous fibrinolysis, and angioplasty.

Statistical analysis

We summarized categorical variables as number (percentage) and numerical variables (continuous variables) as mean and standard deviation (SD). The normality assumptions were assessed for all numerical variables using statistical test. We compared categorical variables using the chi square or Fisher exact test, normally distributed numerical variables with the t test, and other quantitative variables with the Mann-Whitney U test. The differences in baseline characteristics between the genders are expected in the observational studies.

For the adjustment of these differences, a propensity score was generated for the age, BMI, conservative management, diabetes mellitus (DM), hypertension (HTN), dyslipidemia, renal failure and stroke.

Multivariate logistic regression was used to find out the relationship between gender and the different complications considered in this study, adjusting for the generated propensity score. The odds ratios (OR) and estimates with the 95% confidence intervals (CI) were reported for the associations. We assessed model fit using the Hosmer-Lemeshow goodness-of-fit test. We considered a P-value of < 0.05 statistically significant and used SAS® software, version 9.4 (SAS Institute Inc., Cary, NC, USA) for all statistical analyses.

## Results

The data of 900 patients with STEMI - 770 males and 130 females - were analyzed. Females had a higher mean age; 65.7 ± 13.5 for females vs. 58.9 ± 12.8 years for males (p = 0.0001) and BMI was higher in females compared to males and the results were statistically significant (p = 0.0001) as shown in Table 1. The baseline treatment provided to male and female patients with STEMI is shown in Table 2. No statistically significant differences were observed in the treatment provided except for the conservative line of treatment (p = 0.0308). Table 3 presents the results of the statistical analysis for the risk factors for STEMI among male and female patients.

**Table 1:** Age and body mass index of patients with STEMI. STEMI: ST-elevation myocardial infarction

Variables	Male (n = 770) 85.55%	Female (n = 130) 14.44%	p-value
Age mean ± S.D.	58.9 ± 12.8	65.7 ± 13.5	0.0001
Body mass index (BMI)	28.3 ± 5.8	30.5 ± 6.4	0.0001
Mann-Whitney U test is used to calculate the p-value.			

**Table 2:** Baseline treatment provided to the patients. ^ Chi-Square Test; ^^ Fisher's Exact Test.

Baseline treatment	Male	Female	p-value
Percutaneous coronary intervention (PCI)	660 (85.7%)	107 (82.3%)	0.1911^^
Thrombolysis	78 (10.1%)	11 (8.5%)	0.5326^^
Coronary artery bypass surgery (CABG)	72 (9.4%)	8 (6.2%)	0.2257^^
Conservative management	48 (6.2%)	15 (11.5%)	0.0308^^

**Table 3:** Risk factors for complications in STEMI in male and female gender. ^ Chi-Square Test; ^^ Fisher's Exact Test. STEMI: ST-elevation myocardial infarction

Risk factors	Male (N = 770)	Female (N = 130)	p-value
Diabetes mellitus	449 (58.3%)	104 (79.8%)	0.0001 ^
Hypertension	417 (54.1%)	104 (79.8%)	0.0001 ^
Dyslipidemia	472 (61.2%)	92 (70.5%)	0.0103 ^
Previous ischemic heart disease	159 (20.6%)	23 (17.6%)	0.0726 ^
Family history	33 (4.2%)	8 (5.8%)	0.1293 ^
Smoking	373 (48.4%)	12 (8.0%)	0.0001 ^
Renal failure	73 (9.5%)	19 (14.6%)	0.0267 ^
Chronic obstructive pulmonary disease	14 (1.8%)	3 (2.3%)	0.2344 ^^
Stroke	46 (6.0%)	12 (9.2%)	0.0575 ^

The complications observed in the two groups are shown in Table 4. Heart failure was the most common complication in both genders, but its frequency was higher in females than in males with 54 (41.5%) compared to 280 (27%) with significant p-value (p = 0.0010). Mitral regurgitation was the second most frequent complication in both genders, occurring in 41 (31.5%) of females and 169 (21.9%) of males (p = 0.0201). Arrhythmias were diagnosed in 36 (27.7%) of females and 158 (20.5%) in males (p = 0.768). Mortality was higher in females than males 13 (10%) vs. 59 (7.7%), but this difference was not statistically significant (p = 0.3850).

**Table 4:** Complications -Denominator of the percentage is the total number of patients. ^^Chi-square test/**Fisher Exact test is used to calculate the P-value. $ Propensity score adjusted Logistic regression is used to calculate Odds ratio and p-value. aOR*: Adjusted Odds ratio. Male subjects are considered as reference category.

Complications	Male (N = 770)	Female (N = 130)	p-value	aOR	9% CI	p-value
Heart failure	208 (27.0%)	54 (41.5%)	0.0010^^	1.73	(1.149, 2.590)	0.0085^$^
Mitral regurgitation	169 (21.9%)	41 (31.5%)	0.0201^^	1.34	(0.874, 2.081)	0.175
Tricuspid regurgitation	232 (30.1%)	41 (31.5%)	0.8609^	1.03	(0.678, 1.577)	0.875
Arrhythmia	158 (20.5%)	36 (27.7%)	0.0768^^	1.48	(0.951, 2.328)	0.081
Cardiac arrest	104 (13.5%)	20 (15.4%)	0.6009^^	1.25	(0.727, 2.175)	0.411
Cardiogenic shock	78 (10.1%)	14 (10.8%)	0.8610^^	1.23	(0.660, 2.321)	0.506
Died	59 (7.7%)	13 (10%)	0.3850^^	1.16	(0.596, 2.290)	0.651
Heart block	57 (7.4%)	8 (6.2%)	0.5907^^	0.69	(0.316, 1.522)	0.362

## Discussion

Only a few studies have tried to explain the higher mortality in female patients with STEMI as compared to male patients [17-19]. The potential reasons suggested by these authors included a delayed response, differences in the medical attention given to female patients as compared to male patients, or in distinct pathophysiological processes related to the female gender [17]. In our study, we did not find a statistically significant difference in the mortality between the genders, although females had a higher mortality risk than male patients. As described in previous studies, the women in our study had a higher mean age at the time of presentation than men [18,19]. Heart failure was more frequent in female than in male patients. Male patients showed higher levels of myocardial injury marker proteins in the blood, such as CK, CK-MB, and troponin C type I. The frequency of previous coronary interventions and findings on ECG did not significantly differ between the genders.

Similar to other studies, risk factors such as hypertension, diabetes mellitus, and dyslipidemia were more prevalent in females [5,6,12,13]. This higher risk profile might be the cause of the higher incidence rate of complications after STEMI such as heart failure, mitral regurgitation, arrhythmias, and death in females. We believe this is more likely than gender itself being an independent prognostic factor.

The findings in our study are consistent with those of Ng and Lansky [19], Pedersen et al. [20], and, more recently Kanic et al. [21] who also found a correlation between higher age and more frequent comorbidities in women. Females in our study were older than males at the time of diagnosis, which carries a higher risk of comorbidities. The cardio-protective effect of estrogen before menopause is thought to be the cause of this observation [15]. Even though higher rates of smoking have been reported in males, the mortality rate is higher in females [11,14].

In our study, the treatment modalities were similar in both groups except that conservative management was more frequent in females. The high incidence of mitral regurgitation that we found in female patients was also documented by other authors [16]. The significant difference in the incidence of heart failure in our study with 41.5% in females as compared to 27% in males is often attributed to structural differences between the genders [17-20].

Study limitations

As the study design was retrospective in character, the time elapsed between the start of symptoms and beginning of treatment in our patients could not be determined. Moreover, the results of the study is based on one center thus we could not collect a larger sample.

## Conclusions

In conclusion, despite the advances in the technology and the use of novel reperfusion therapies, females were associated with poorer outcomes after adjustment of the baseline characteristics and risk factors. In other words, heart failure, mitral regurgitation, and arrhythmias were higher in females with significant p values. Further studies are needed to support the results of this research as it was a single center study.
